# Changes in Activity and Community Composition Shape Bacterial Responses to Size-Fractionated Marine DOM

**DOI:** 10.3389/fmicb.2020.586148

**Published:** 2020-11-20

**Authors:** Marta M. Varela, Tamara Rodríguez-Ramos, Elisa Guerrero-Feijóo, Mar Nieto-Cid

**Affiliations:** ^1^Centro Oceanográfico de A Coruña, Instituto Español de Oceanografía (IEO), A Coruña, Spain; ^2^Laboratorio de Geoquímica Orgánica, Instituto de Investigaciones Marinas (CSIC), Vigo, Spain

**Keywords:** bacterial diversity, amplicon sequencing variants, flow cytometry, dissolved organic matter, DOM optical properties, tangential ultrafiltration, size-fractionated DOM, remineralization

## Abstract

To study the response of bacteria to different size-fractions of naturally occurring dissolved organic matter (DOM), a natural prokaryotic community from North Atlantic mesopelagic waters (1000 m depth) was isolated and grown in (i) 0.1-μm filtered seawater (CONTROL), (ii) the low-molecular-weight (<1 kDa) DOM fraction (L-DOM), and (iii) the recombination of high- (>1 kDa) and low-molecular-weight DOM fractions (H + L-DOM), to test the potential effect of ultrafiltration on breaking the DOM size continuum. Prokaryotic abundance and leucine incorporation were consistently higher in the H + L-DOM niche than in the L-DOM and CONTROL treatments, suggesting a different interaction with each DOM fraction and the disruption of the structural DOM continuum by ultrafiltration, respectively. Rhodobacterales (Alphaproteobacteria) and Flavobacteriales (Bacteroidetes) were particularly enriched in L-DOM and closely related to the colored DOM (CDOM) fraction, indicating the tight link between these groups and changes in DOM aromaticity. Conversely, some other taxa that were rare or undetectable in the original bacterial community were enriched in the H + L-DOM treatment (e.g., Alteromonadales belonging to Gammaproteobacteria), highlighting the role of the rare biosphere as a seed bank of diversity against ecosystem disturbance. The relationship between the fluorescence of protein-like CDOM and community composition of populations in the H + L-DOM treatment suggested their preference for labile DOM. Conversely, the communities growing on the L-DOM niche were coupled to humic-like CDOM, which may indicate their ability to degrade more reworked DOM and/or the generation of refractory substrates (as by-products of the respiration processes). Most importantly, L- and/or H + L-DOM treatments stimulated the growth of unique bacterial amplicon sequence variants (ASVs), suggesting the potential of environmental selection (i.e., changes in DOM composition and availability), particularly in the light of climate change scenarios. Taken together, our results suggest that different size-fractions of DOM induced niche-specialization and differentiation of mesopelagic bacterial communities.

## Introduction

Dissolved organic matter (DOM) is a complex mixture of compounds that constitutes a major source of carbon and energy in aquatic ecosystems (Hansell et al., 2009). Most DOM in the oceans originates from phytoplankton, either via extracellular release or via zooplankton grazing and viral lysis (Buchan et al., 2014). A significant fraction of this DOM is remineralized in sunlit surface waters, whereas a variable fraction is exported into the dark ocean realm (Buchan et al., 2014) fueling deep sea microbial communities (Arístegui et al., 2009). The size-reactivity continuum model hypothesizes how the bioreactivity of natural organic matter decreases, in general, along a continuum of size, diagenetic alteration and age, giving rise to a net flow of organic carbon from larger to smaller size classes with increasing decomposition (Benner and Amon, 2015). Besides, this reactivity is strongly dependent on environmental variables, such as inorganic nutrients or temperature (Arnosti et al., 2011). Hence, marine heterotrophic bacteria utilize DOM differentially depending on both chemical composition and molecular size (Amon and Benner, 1996; Nagata, 2008). Considerable effort aimed at describing DOM has focused on characterizing the high-molecular-weight fraction of DOM (>1 kDa), that can contain both refractory compounds, resistant to biological or chemical processes (e.g., humic substances), and/or labile material that can be rapidly utilized by marine bacteria (Amon and Benner, 1996; Ogawa and Tanoue, 2003). The majority of marine DOM is found below 1000 m depth, where it is transported by deep ocean circulation and exposed to very low degradation rates (Barber, 1968; Benner, 2002; Hansell, 2013). Results from degradation experiments indicate that most of this dark ocean DOM falls within the low-molecular-weight fraction (<1 kDa) of DOM (Amon and Benner, 1996), originated by microbial processes that make this DOM resistant to further degradation by altering the molecular structure (Ogawa et al., 2001). Nevertheless, compounds like urea and free amino acids, which are also part of the low-molecular-weight DOM, are known to be highly labile (Hansell and Carlson, 2015). Rapid uptake by microbes might likely help to explain the low concentrations of such compounds in deep seawater. However, the reactivity of DOM does not only depend on the size and chemical characteristics of the organic compounds, but should be also dependent on the composition of the consumer community.

In the oceans, water masses are characterized by hydrographic signatures and associated microbial communities (Galand et al., 2009; Agogué et al., 2011). Yet, much remains to be learned about whether activity and community compositional variances in different water masses have consequences for organic matter degradation. A previous work in the deep North Atlantic Ocean indicated a bacterial community structure shaped not only by depth-related physicochemical features but also by DOM quality (Dobal-Amador et al., 2016). Later studies in the same area found associations of some specific bacterial taxa with particular DOM optical signatures under *in situ* conditions, suggesting the relation between SAR324 and the degradation of DOM in deep waters, and/or the connection between SAR202 and SAR406 with refractory DOM compounds (Guerrero-Feijóo et al., 2017). However, it remains unknown which microbial groups are relevant to the degradation of different size fractions of naturally occurring mesopelagic DOM.

Mesopelagic environments are characterized by more stable physical conditions than those found in surface waters (Costello and Breyer, 2017), while the composition of organic constituents display high variability and it is linked to microbial community structure (Kaiser and Benner, 2012; Guerrero-Feijóo et al., 2017). Upwelling systems are sites of enhanced primary production and organic matter export. In particular, the study region in the Atlantic Iberian margin (from 43°N, 9°W to 43°N, 15°W) is a dynamic area characterized by seasonal upwelling pulses, which support both the offshore export and sinking fluxes of organic matter (Teira et al., 2003; Álvarez-Salgado et al., 2006; Lønborg et al., 2015). Mixing of different water masses reaches down to the mesopelagic layer that flows northwards along the western Iberian Peninsula (Ruiz-Villarreal et al., 2006) leading to organic matter fluxes and consequently affecting to the abundance and composition of the microbial communities inhabiting the dark ocean (Guerrero-Feijóo et al., 2017). Therefore, it represents an ideal area to test the hypothesis that different size fractions of natural DOM will select bacterial communities with different activity, diversity and/or community composition, linked to changes in the composition of DOM as far as degradation occurs. Thus, we set up an experimental approach to investigate the effect of size-fractionated (presumably with different composition) naturally occurring DOM on: (i) prokaryotic abundance and activity; and (ii) bacterioplankton diversity and community composition; and their links to (iii) optical indices of the available DOM as far as it is degraded and modified by the biological community.

## Materials and Methods

### Sampling and Experimental Set-Up

Seawater from the mesopelagic layer (1000 m depth) was collected on the 7th August 2014 during the MODUPLAN cruise, on board R/V Sarmiento de Gamboa. Seawater was sampled at St. 11 (43°N, 10°W) using 12-L Niskin bottles mounted on a CTD-rosette sampler and collected in acid-washed polycarbonate carboys (Nalgene). Firstly, 75 L of the seawater sample were filtered through 0.1 μm membrane filters (142 mm diameter, Supor-100, Pall Corporation) to completely remove bacteria. Subsequently, 50 L of the filtrate were filtered again using a tangential flow ultrafiltration system with a 1 kDa ultrafiltration membrane (GE Series, GE Power and Water), splitting (size-fractionating) the sample into high (>1 kDa, final volume 1.75 L) and low (<1 kDa, final volume 48.25 L) molecular weight fractions. The concentration factor (CF = sample volume/retentate volume) was quite low (∼28) to ensure a pure low-molecular-weight fraction (Martínez-Pérez et al., 2017). Three different base treatments were performed: (i) a CONTROL treatment using 0.1 μm filtered seawater (which is representative of all-size DOM without further manipulation); (ii) a L-DOM treatment using the 1 kDa filtered seawater (which is representative of low-molecular-weight DOM; <1 kDa) and (iii) a H + L-DOM treatment that was prepared by recombining the 1 kDa filtered seawater (<1 kDa; low-molecular-weight DOM) and the retentate seawater (<0.1 μm and >1 kDa, high-molecular-weight DOM) in the same proportion as the tangential flow separation (which is also representative of all-sizes DOM). This last treatment was performed to take into account the possible effect of the ultrafiltration processes on breaking the DOM reactivity-size continuum. A fraction of the original ambient seawater was filtered using 0.6 μm filters (142 mm diameter, polycarbonate hydrophilic membranes, ipPORE^TM^ Track Etched Membrane). This filtrate containing the natural bacterial community was used as the inoculum (dilution 1:10) for all the treatments. The three treatments were prepared as 7-L microcosms in triplicate and were kept at controlled *in situ* temperature (10°C) in the dark to simulate the environmental conditions of the mesopelagic waters. Experimental incubations were monitored daily over 6 days. Due to the low DOM concentrations and to avoid contamination we did not filter the samples before the measurements of the optical properties of DOM, so they actually include the DOM but also the bacteria growing in each treatment. Microbial growth was assessed daily by determining prokaryotic abundance and leucine incorporation until the communities reached the stationary phase. A volume of 2–4 L was filtered at the end of the experiment to assess community composition in the different treatments. The filters were flash-frozen in liquid nitrogen and stored at −80°C until DNA extraction.

### Inorganic Nutrients

Water samples for nutrient salts (nitrate, nitrite, and phosphate) analysis were collected at the onset and the end of the experiment. Water samples were frozen on board until measured in the home laboratory using a QuAAtro auto-analyzer from SEAL Analytical. The protocols from SEAL analytics Q-126-12 and Q-104-09 were used for nitrate and nitrite, and Q-125-12 for phosphate concentration analysis, respectively (Coverly et al., 2012).

### DOM Optical Properties

DOM optical properties were measured on board at the beginning and at the end of the experiment, by pouring small aliquots of sample (5–25 mL) directly into the corresponding optical measurement cell. On the one hand, fluorescence intensity was measured with a Perkin Elmer LS55 following Nieto-Cid et al. (2006) at two excitation/emission wavelengths pairs: (i) 320 nm/410 nm (peak M), characteristic of marine humic-like substances, and (ii) 280 nm/350 nm (peak T), characteristic of protein-like molecules. Samples were calibrated against quinine sulfate and results are given in quinine sulfate units (QSU) by subtracting fresh Milli-Q water. On the other hand, the absorption spectra of chromophoric DOM were acquired on a Beckman Coulter DU800 spectrophotometer equipped with quartz cells (10 cm length). Spectral scans were collected from 250 to 700 nm providing the following indices (Green and Blough, 1994): (i) a254 (absorption coefficient at 254 nm, used as a proxy for dissolved organic carbon; Lønborg and Álvarez-Salgado, 2014); a340 (absorption coefficient at 340 nm) and a365 (absorption coefficient at 365 nm), and (ii) s275-295 (slope of the absorption spectrum between 275 and 295 nm, providing information on shifts in molecular mass and DOM aromaticity; Helms et al., 2008; Catalá et al., 2018). The differences between the absorption coefficients lay on the nature of the colored DOM, as the intensification of the conjugation/aromaticity increases with the absorption wavelength (Stedmon and Nelson, 2015; Catalá et al., 2018). Thus, absorption coefficients at wavelength larger than 300 nm would only gather information for complex/aromatic molecules and would not be related to relatively simple compounds, which are related instead to a254.

### Total Prokaryotic Abundance

Total prokaryotic abundance (PA) was determined by flow cytometry as previously described by Gasol et al. (1999). Briefly, water samples (1.8 mL) were preserved with 1% paraformaldehyde plus 0.05% glutaraldehyde (final concentration), shock-frozen in liquid nitrogen for 5 min and stored at −80°C until further analysis. Samples were thawed and stained with Syto13 for 10 min in the dark. Fluorescent latex beads (approximately 1 × 10^5^ mL^–1^; Molecular Probes, 12 Invitrogen, Carlsbad, CA) were added as internal standard. Prokaryotic cells were counted using a FACSCalibur flow cytometer (Becton Dickinson, Franklin Lakes, NJ, United States) according to their signature in right angle light scatter and green fluorescence. Two types of cells, low nucleic acid (LNA) and high nucleic acid (HNA) content, were differentiated. The proportion of HNA bacteria from the prokaryotic community (%HNA) was calculated as the number of HNA cells divided by total (LNA + HNA) Syto-13 cell counts (Morán and Calvo-Díaz, 2009).

### Bacterial Membrane Integrity

We followed Grégori et al. (2001) to evaluate cell membrane integrity using nucleic acid double staining (NADS) with SYBR Green I (Molecular Probes, ref. S-7563) and propidium iodide (PI; Sigma, ref. P-4170). Water samples (0.4 mL) were stained with 1× SYBR Green I and 10 μg mL^–1^ PI for 15 min in the dark at room temperature. Subsequently to the addition of the beads solution as internal standard, the samples were run in a FACSCalibur flow cytometer. Cells impermeable to PI and cells permeable to PI (i.e., cells with intact and damaged membranes, respectively) were distinguished according to their signature in red and green fluorescence. The terms “live” and “dead” were subsequently used to designate cells with intact and damaged membranes, respectively. To obtain a coherent dataset, the percentage of live cells (%live) was calculated as the number of live cells divided by the sum of live and dead cells, rather than to total bacteria previously quantified by Syto13-staining (Morán and Calvo-Díaz, 2009).

### Actively Respiring Bacterial Cells

We used the redox dye 5-Cyano-2,3-di-(*p*-tolyl)tetrazolium chloride (CTC, Polysciences, ref. 19292) to identify actively respiring cells. Water samples (0.25 mL) were incubated with CTC (5 mmol L^–1^, final concentration) for *c*. 90 min in dark at *in situ* temperature as described in Morán and Calvo-Díaz (2009). Subsequently, the samples were immediately run through the flow cytometer, and the CTC + cells were identified according to their signature in red *versus* orange fluorescence. The proportion of CTC + cells (%CTC +) was calculated for each sample as the number of CTC + cells versus the total bacterial counts estimated with Syto-13 (Morán and Calvo-Díaz, 2009).

### Prokaryotic Leucine Incorporation

Samples to measure leucine incorporation rates by heterotrophic prokaryotes were inoculated with 5 nM ^3^H-leucine (final concentration, specific activity 160 Ci mmol^–1^) and incubated in the dark at *in situ* temperature for 4–6 h depending on the expected activity. Subsequently, TCA (5% final concentration) was added to the samples. The samples were pelleted by centrifugation (12350 g, 10 min) and washed with 1 mL of 5% TCA (Kirchman et al., 1985). Afterward, 1 mL of scintillation cocktail was added to the pellets and after 18 h and the radioactivity was determined in a scintillation counter (TRI-CARB Liquid Scintillation Counter Perkin Elmer). The mean disintegrations per minute (DPM) of the blanks were subtracted from the mean DPM of the respective samples. The obtained disintegrations per minute (DPMs) were converted to leucine incorporation rates (Leu incorp., pmol leu h^–1^ L^–1^). The cell-specific activity (pmol leu cell^–1^ h^–1^) was estimated dividing leucine incorporation rate by prokaryotic abundance.

### DNA Extraction, Sequencing and Bioinformatics

Samples for DNA analysis were collected in the three different treatments at the end of the experiment (day 6). A volume of 2 L of water was filtered onto a 0.2 μm polycarbonate filter (Millipore) and the filters were subsequently stored at −80°C until further analysis. DNA extraction was performed with PowerSoil DNA Isolation Kit (MO BIO) according to manufacturer protocol. Subsequently, the 9 DNA extracts were sent to the Research and Testing Laboratory (Lubbock, TX, United States)^1^ for 454-pyrosequencing with the primers 341F (CCTACGGGNGGCWGCAG) and 805R (GACTACHVGGGTATCTAATCC) (Herlemann et al., 2011), generating amplicons spanning the V3 to V4 regions of the bacterial 16S rRNA gene. Exact amplicon sequence variants (ASVs) were differentiated by using the package *dada2* (Callahan et al., 2016) implemented in R (R Core Team, 2019), which resolves ASVs inferring exact variants up to 1 nucleotide of difference. Sequences were aligned against SILVA 132 16S rRNA database (Quast et al., 2012) as reference. Finally, singletons (ASVs found only once in the final ASV table) were excluded, as they have been shown to likely be the result of PCR or sequencing errors (Huse et al., 2010). Pyrotag sequences have been deposited in the National Center for Biotechnology Information (NCBI) Sequence Read Archive (SRA) under PRJNA385510 BioProject number.

Given the small differences in the number of reads among samples (average difference 397 ± 49 reads), we retained all our reads (McMurdie and Holmes, 2014) to avoid losing diversity. ASV richness was calculated with the function *estimateR* and Shannon diversity index was computed with the function *diversity*, both included in the *vegan* package (Oksanen et al., 2012) in R (R Core Team, 2019). Venn diagrams were built using the R package *VennDiagram* (Chen and Boutros, 2011). Fold change was estimated as the ratio between the relative abundance (%) of a given ASV or taxonomic group in the L-DOM and/or H + L-DOM treatment with respect to its relative abundance in the CONTROL treatment. Because some ASVs were absent in CONTROL (relative abundance = 0), relative abundances in all treatments were incremented in one unit, adding up 1, allowing Fold Change to be computed while keeping proportions between the compared relative abundances. To improve the interpretation of data, this expression was transformed to “Fold Change – 1,” so that decreasing responses compared against CONTROL (Fold Change between 0 and 1) are expressed as negative values.

### Statistical Analysis

Normality was tested with the Shapiro-Wilk test. For comparative analysis of the different variables among the three treatments, ANOVA and post host pairwise *t*-test were performed. These statistical analyses were performed with SPSS software.

The SIMPER analysis, quantified with the function *simper* included in the *vegan* package (Oksanen et al., 2012) in R (R Core Team, 2019), allows to identify ASVs that are likely to be the major contributors to the dissimilarity found between each pair of groups (Clarke, 1993) (L-DOM *versus* H + L-DOM, CONTROL *versus* L-DOM, and CONTROL *versus* H + L-DOM).

Redundancy analysis (RDA) was performed to test the relationship among shifts in DOM optical properties (peak M, peak T, a254, a340, a365, s275-295), single-cell physiological properties (%CTC + and%live), and bacterioplankton community composition in the different treatments (Ramette, 2007). PERMANOVA was used to test for differences in prokaryotic community structure among treatments. These statistical analyses were performed and visualized by XLSTAT software^2^ (Addinsoft, New York, NY, United States).

## Results

### Inorganic Nutrients and Optical Characterization of DOM

At the beginning of the experiment, averaged (mean ± SD) concentrations of NO_3_^–^ + NO_2_^–^ showed similar values in the three treatments (13.70 ± 0.04, 13.74 ± 0.02 and 13.63 ± 0.07 μM in CONTROL, H + L- and L-DOM, respectively, ANOVA *p* > 0.05). Also, the mean PO_4_^3–^ initial concentration was similar in all treatments (0.99 ± 0.03, 0.97 ± 0.02 and 0.96 ± 0.03 μM in CONTROL, L- and H + L-DOM, respectively, ANOVA *p* > 0.05). Changes in PO_4_^3–^ concentration were not significant after the 6-day incubation, whereas the inorganic nitrogen concentration (NO_3_^–^ + NO_2_^–^) showed different dynamics in the three treatments. The NO_3_^–^ + NO_2_^–^ concentration was lower in CONTROL and H + L-DOM than in L-DOM treatment after 6 days of experiment (on average, 13.61 ± 0.25, 13.47 ± 0.05 and 13.81 ± 0.02 μM in CONTROL, H + L- and L-DOM, respectively). Indeed, the concentration of inorganic nitrogen exhibited a significant decrease (mean ± SD) by −0.27 ± 0.09 (*t*-test, *p* < 0.05) μmol kg^–1^ in H + L-DOM treatment, whereas it increased significantly by 0.18 ± 0.01 μmol kg^–1^ (*t*-test, *p* < 0.05) in L-DOM treatment (Supplementary Table 1). CONTROL displayed a slight decrease in inorganic nitrogen concentration between day 0 and day 6 (−0.09 ± 0.03), although those difference was not statistically significant (*t*-test, *p* > 0.05).

Changes in the optical properties of DOM (mean ± SD) for each treatment during the experimental period are summarized in Table 1 (see also Supplementary Table 2). Peak M did not display significant changes in any of the treatments between day 0 and day 6 (*t*-test, *p* > 0.05). Peak T significantly increased from day 0 to 6 by 0.7 ± 0.4 and 0.9 ± 0.4 QSU in CONTROL (*t*-test, *p* < 0.05) and H + L-DOM treatments (*t*-test, *p* < 0.05), respectively. Absorption coefficient a254 significantly decreased with time in the three treatments (−0.05 ± 0.02, −0.10 ± 0.06 and −0.07 ± 0.02 m^–1^; *t* test, *p* < 0.05; in CONTROL, H + L- and L-DOM treatments, Table 1). Similarly, a340 and a365 decreased in the three treatments by the end of the experiment (Table 1), although these differences were only statistically significant in CONTROL and L-DOM treatments (*t*-test, *p* < 0.05). Conversely, the absorption slope (s275-295) increased over the time course of the experiment, although not significant differences were found between sampling times (*t*-test, *p* > 0.05).

**TABLE 1:** Variation of the average (mean ± SD) DOM optical properties between day 0 and day 6 in the three treatments: 0.1-μm filtered seawater (CONTROL), the recombination of high- (>1 kDa) and low- (<1 kDa) molecular-weight DOM fractions (H + L-DOM) and the low-molecular-weight DOM fraction (L-DOM).

	**CONTROL**	**H + L-DOM**	**L-DOM**
Peak M (QSU)	0.01 ± 0.04	−0.02 ± 0.06	0.00 ± 0.06
Peak T (QSU)	0.7 ± 0.4	0.9 ± 0.4	0.1 ± 0.6
a254 (m^–1^)	−0.05 ± 0.02	−0.10 ± 0.06	−0.07 ± 0.02
a340 (m^–1^)	−0.02 ± 0.01	−0.02 ± 0.06	−0.08 ± 0.02
a365 (m^–1^)	−0.02 ± 0.00	−0.03 ± 0.03	−0.03 ± 0.02
s275-295	0.0004 ± 0.0004	0.002 ± 0.004	0.004 ± 0.004

*Abbreviations: Peak M (QSU), marine humic-like substances; Peak T (QSU), protein-like substances; a254 (QSU), absorption coefficient at 254 nm; a340 (m^–1^), absorption coefficient at 340 nm; a365 (m^–1^), absorption coefficient at 365 nm; s275-295, the spectral slope between 275 and 295 nm. See also Supplementary Table 2 for further details.*

### Bulk and Single-Cell Microbial Properties

**FIGURE 1:**
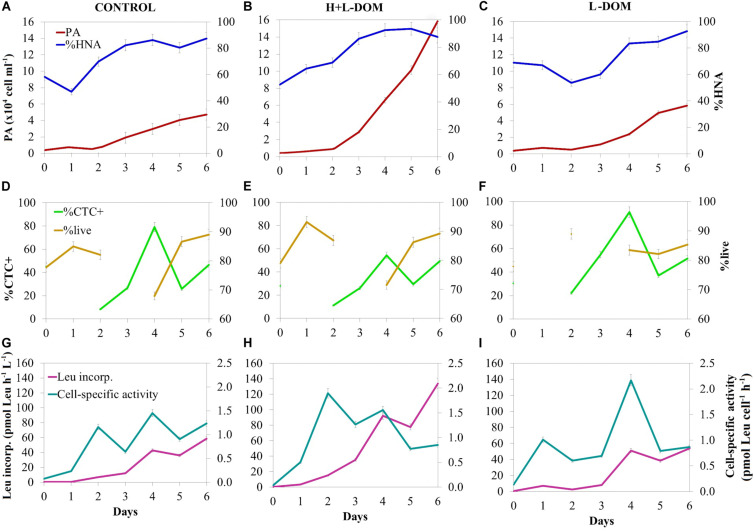
Time course of bulk and single-cell microbial properties during the experiment. Prokaryotic abundance (PA) and % of high nucleic content (%HNA) present in panel **(A)** CONTROL, **(B)** H + L-DOM, and **(C)** L-DOM. The % of actively respiring cells (% CTC +) and % of live cells (%live) in panel **(D)** CONTROL, **(E)** H + L-DOM and **(F)** L-DOM. Leucine incorporation rate and cell-specific activity in panel **(G)** CONTROL, **(H)** H + L-DOM, and **(I)** L-DOM. See Table 1 for treatments definition.

The prokaryotic abundance (PA) was similar in CONTROL, H + L-DOM and L-DOM treatments at the beginning of the experiment (0.70 ± 0.24 × 10^4^, 0.43 ± 0.11 × 10^4^, and 0.37 ± 0.06 × 10^4^ cells mL^–1^, respectively; ANOVA *p* > 0.05). PA increased exponentially in the three treatments until day 6 (Figures 1A–C). CONTROL and L-DOM treatments yielded significantly lower PA (4.73 ± 0.28 × 10^4^ and 5.84 ± 1.03 × 10^4^ cell mL^–1^, respectively) as compared to H + L-DOM (15.80 ± 3.80 × 10^4^ cell mL^–1^) (*t*-test, *p* < 0.05) at the end of the experiment. In the three treatments, cells with high nucleic acid content (%HNA) dominated the community throughout the time span of the experiment (Figures 1A–C), with relative abundance >50% in most of the samples. Besides, live microbial cells (%live) were more abundant (>50%) than dead cells throughout the time span of the experiment (*t*-test, *p* < 0.05). Significant differences in live microbial cells (%live) were not found among the three treatments (ANOVA, *p* > 0.05), although they reached higher average values in CONTROL (∼70%) and H + L-DOM (∼80%) than in L-DOM (∼60%) (Figures 1D–F). The percentage of actively respiring cells (%CTC +) represented a variable fraction of the total community throughout the time span of the experiment, and no significant differences were found among the three treatments (ANOVA, *p* > 0.05) (Figures 1D–F).

Leucine incorporation rate (Leu incorp.) increased over time in all treatments and showed significantly 3-fold higher rates (*t*-test, *p* < 0.05) on day 6 in H + L-DOM (1.34 ± 0.30 × 10^2^ pmol Leu L^–1^ h^–1^) as compared to CONTROL and L-DOM (0.59 ± 0.07 × 10^2^ and 0.54 ± 0.17 × 10^2^ pmol Leu L^–1^ h^–1^, respectively) (Figures 1G–I). Cell-specific activity (Leu incorp. per cell) generally increased in all treatments from the beginning of the experiment to day 4 to subsequently slightly decrease down to ∼1 pmol Leu cell^–1^ h^–1^ in all treatments at the end of the experiment (Figures 1G–I).

### Bacterial Richness, Diversity and Community Composition Growing on Different DOM

A total of 236 ASVs were detected in the three treatments after the incubation period. Significant differences were found in observed ASV richness (S_*obs*_) among the three treatments (ANOVA, *p* < 0.05). H + L-DOM treatment displayed significantly higher S_*obs*_ than L-DOM (Tukey’s test, *p* < 0.05), while L-DOM showed S_*obs*_ significantly lower than CONTROL treatment (Tukey’s test, *p* < 0.05). Conversely, we did not find significant differences in the Shannon index among the three treatments (ANOVA, *p* > 0.05) (Table 2).

**TABLE 2:** Mean (±SD) observed ASV richness (S_*obs*_), and Shannon diversity index of bacterial communities growing in the three treatments (CONTROL, H + L-DOM and L-DOM, see Table 1 for treatments definition) at the end of the experiment (day 6).

**Treatment**	**S_*Obs*_**	**Shannon**
CONTROL	35.6 ± 0.8	3.0 ± 0.2
H + L-DOM	44 ± 2.6	3.3 ± 0.1
L-DOM	33.6 ± 1.4	2.9 ± 0.1

**FIGURE 2:**
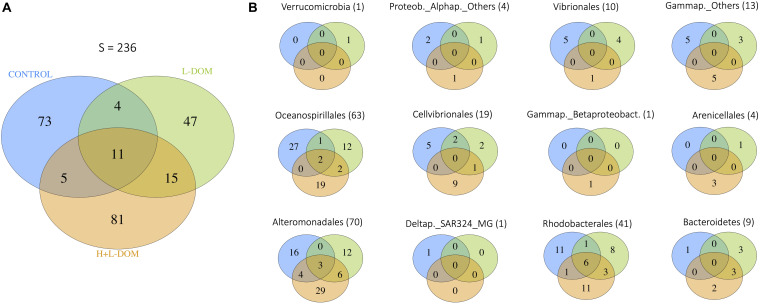
Venn diagrams showing **(A)** the number of ASVs shared among the CONTROL, H + L-DOM and L-DOM treatments, as well as unique ASVs for each treatment, for the whole bacterial community; and **(B)** the number of ASVs shared among CONTROL, H + L-DOM and L-DOM, as well as unique ASVs for each treatment, for specific bacterial Orders. In brackets, the total number of ASVs belonging to each Order is shown. See Table 1 for treatments definition.

After 6 days of incubation, a relatively narrow phylogenetic bacterial community was found in all treatments as compared to the original community (see Supplementary Figure 1). However, treatments were remarkably different in terms of community composition among each other (Figure 2). The number of ASVs shared among the three treatments (11 ASVs, <10% of total ASV richness), as well as between pairs of treatments, was relatively low (Figure 2A). Consequently, communities growing under the different treatments were composed mostly by unique ASVs (i.e., they were present only in one of the treatments), which belonged mainly to the Orders Oceanospirillales, Alteromonadales and Rhodobacterales (Figure 2B and Supplementary Table 3). SIMPER analysis quantified the contribution of ASVs to the dissimilarity found between pairs of treatments (Supplementary Table 4). It showed that 21 ASV belong to 8 different genera [*Shimia* (ASV23, ASV25, ASV24, ASV26, ASV27), *Shewanella* (ASV115, ASV116, ASV117, ASV118), *Thalassotalea* (ASV73, ASV74, ASV75), *Lentibacter* (ASV12, ASV13), *Olephilus* (ASV151), Vibrio (ASV213), *Marinobacter* (ASV86, ASV87, ASV91) and *Sacharospirillaceae* (ASV183, ASV184)], explained more than 50% of the variation between DOM-treatments (L- and H + L-DOM). The greatest contribution to differences between CONTROL and both L- and H + L-DOM treatments was due to *Lentibacter* (ASV12), *Shewanella* (ASV115), *Sacharospirillaceae* (ASV183), *Shimia* (ASV23, ASV24) and *Thalassotalea* (ASV73) which explained between 13.89 and 21.39%, respectively.

**FIGURE 3:**
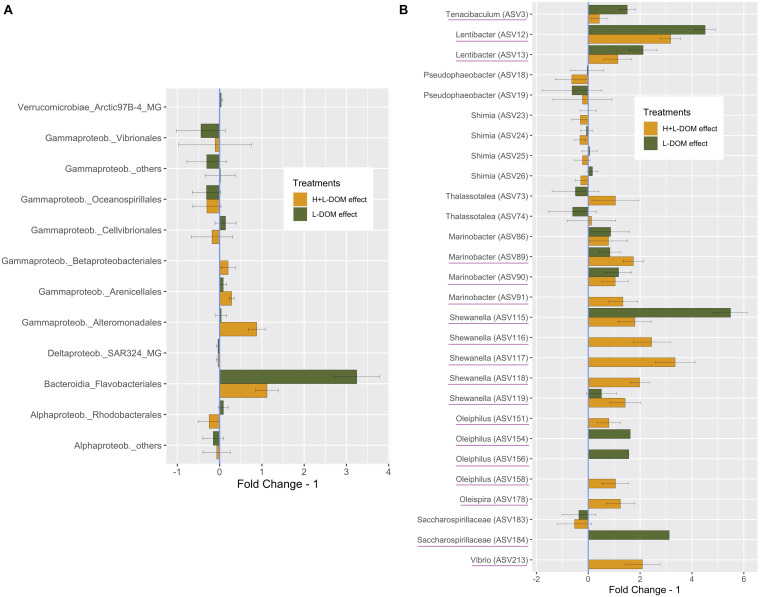
Average “Fold Change – 1,” based on the average relative abundance under H + L-DOM and/or L-DOM compared against CONTROL, at **(A)** Order-level **(B)** individual ASV-level (only ASVs with a difference in relative abundance >1% compared against CONTROL are shown). “Fold Change – 1” = 0 indicates no difference in relative abundance in a treatment compared to CONTROL, while negative values denote decreasing relative abundances compared against CONTROL. Underlined ASVs are those which were absent in CONTROL but were present (relative abundance >0) in any or both DOM-treatments. Bars denote the propagated standard errors. Flavobacteriales, Rhodobacterales and Alteromonadales were significantly more enriched in H + L-DOM than in L-DOM (*t*-test, *p* < 0.05). See Table 1 for treatments definition.

In order to investigate the principal responders to the size-fractionated DOM treatments, we calculated the average fold change in relative abundance of ASVs growing in H + L-DOM and/or L-DOM compared against CONTROL treatment (Figure 3). At Order level (Figure 3A), ASVs belonging to Flavobacteriales and Rhodobacterales experimented significantly higher enrichment in L-DOM than in H + L-DOM treatment (*t*-test, *p* < 0.05). Also, ASVs belonging to Cellvibrionales were more enriched in L-DOM than in H + L-DOM treatment, although the difference in fold change for these ASVs between the two treatments was not statistically significant (*t*-test, *p* > 0.05). Conversely, ASVs belonging to Alteromonadales were significantly more enriched in H + L-DOM than in L-DOM (*t*-test, *p* < 0.05). In the same way, Betaproteobacteriales, and to a lesser extent Arenicellales, also displayed higher fold change in H + L- than in L-DOM, although the differences in relative abundance of ASVs belonging to this order were not statistically significant (*t*-test, *p* > 0.05).

Interestingly, Rhodobacterales, Cellvibrionales and Gammaproteobacteria_others presented opposite fold changes in L-DOM and H + L-DOM treatments. While Rhodobacterales and Cellvibrionales were enriched in L-DOM, these orders decreased in relative abundance in H + L-DOM. Conversely, H + L-DOM treatment stimulated the growth of ASVs belonging to Gammaproteobacteria, while they decreased under the L-DOM effect. At individual ASV-level (Figure 3B), ASVs belonging to *Tenacibaculum* (ASV3), *Lentibacter* (ASV12 and ASV13), *Shewanella* (ASV115), *Oleiphilus* (ASV154 and ASV156) and Saccharospirillaceae (ASV184) showed greater enrichments in L-DOM (fold change >2) than in H + L-DOM. Conversely, ASVs belonging to *Thalassotalea* (ASV73 and ASV74), *Marinobacter* (ASV89 and ASV91), *Shewanella* (ASV116, ASV117, ASV118, and ASV119), *Oleiphilus* (ASV158), *Oleispira* (ASV178), and *Vibrio* (ASV213) displayed higher enrichments in H + L-DOM (fold change >2) than in L-DOM treatment. Finally, some ASVs belonging to *Shimia* and *Thalassotalea* showed opposite fold changes in H + L-DOM and L-DOM treatments. While *Shimia* (ASV25, ASV26) increased in L-DOM, those ASVs showed a decrease when growing at the expense of H + L-DOM. Conversely, *Thalassotalea* (ASV74, ASV75) showed enrichment in H + L-DOM treatment, whereas those ASVs decreased in L-DOM.

### Links Between the Activity and Community Composition of Bacteria Growing on Different DOM Size-Fractions and Their Optical Properties

**FIGURE 4:**
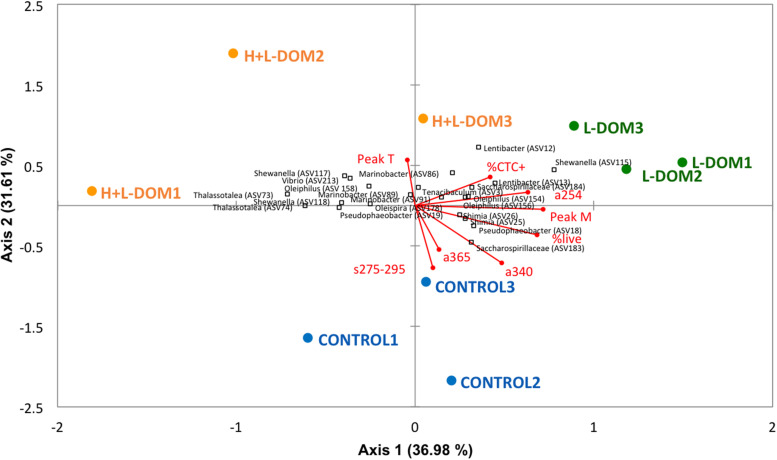
Redundancy Analysis (RDA) showing the vectors for the different optical indices of the DOM and for the bulk bacterial parameters along with the main bacterial phylotypes related to them. Blue circles, green squares, and orange squares represent respectively the replicates of CONTROL, H + L-DOM and L-DOM treatments. The direction and length of the vectors indicate the increase of the different variables. See Table 1 and Figure 1 for abbreviations of DOM optical properties and microbial properties.

Significant differences were found in community composition among the three treatments (Permutational multivariate analysis of variance, PERMANOVA, *p* < 0.05). Redundancy analysis (Figure 4) axes 1 and 2 explained ∼37 and ∼32% of the variability of the bacterial communities, respectively. The proportion of actively respiring cells (%CTC +) and a254 were positively correlated with both axes. Peak T was negatively correlated with axis 1 and positively with axis 2. Peak M, %live, a340, a365 and s275-295 were positively correlated with axis 1 but negatively with axis 2. RDA indicated that s275-295, a365 and a340 were the main DOM indices differentiating CONTROL *versus* H + L-DOM and L-DOM communities. H + L-DOM communities, particularly some Alteromonadales (such as *Thalassotalea sp*., *Shewanella sp*., and *Marinobacter*), Oceanospirillales (such as *Oleiphilus*), and Vibrionales (such as *Vibrio*) were associated to protein-like substances (peak T) and%CTC + cells. In contrast, a254, peak M and%live were associated with L-DOM communities, particularly with members of Rhodobacterales (such as *Lentibacter* sp and *Pseudobacter* sp.), Flavobacteriales (such as *Tenacibaculum*), and Oceanospirillales (such as Sacharospirillaceae and *Oleiphilus*).

## Discussion

DOM in marine environments is composed by a heterogeneous size-reactivity continuum of compounds, including low- and high-molecular-weight substrates, influencing the microbial abundance and composition as well as their metabolic activity. Several studies have shed light on the bacterial utilization of the high- and low-molecular-weight fractions of natural DOM, most of them focusing on the relationship of different DOM fractions with microbial bulk parameters (i.e., bacterial abundance and production) (e.g., Amon and Benner, 1994; Khodse and Bhosle, 2011; Amon, 2016) and some on the identity of Bacteria cycling DOM (i.e., Covert and Moran, 2001; Sharma et al., 2014; Balmonte et al., 2019). On the other hand, degradation processes might shape the size continuum distribution of organic matter and the nature of the small dissolved organic molecules that persist in the ocean (Benner and Amon, 2015). Despite these advances in disentangling the interactions between bacterial and DOM size fractions, so far there are no studies about the effect of different size-fractionated natural DOM on bacterial activity, diversity and community composition, concomitant with changes in the DOM composition which follow microbial degradation of naturally occurring DOM in the dark ocean.

### Changes in Bacterial Bulk Properties and Links With DOM Optical Indices in Response to Size-Fractionated DOM Effect

A detailed analysis of the DOM optical indices during our experiment revealed that a254, used as a proxy for DOC concentrations (Lønborg and Álvarez-Salgado, 2014; Catalá et al., 2018), decreased significantly over the time course of the experiment. The decline of this index, together with an increase in inorganic nitrogen concentration in the L-DOM incubations, confirmed the bioavailability of a fraction of the DOM in this treatment. Besides, the increase in the spectral absorption slope s275-295 (proxy for the average molecular weight of the DOM; Helms et al., 2008) over the time course of the experiment indicated a decreased of the average molecular weight of DOM during the incubation period. This fact suggests that the relatively higher-molecular-weight DOM available in both L- and H + L-DOM treatments was likely preferentially used by bacteria, in accordance to Benner and Amon (2015).

Additionally, our results confirm that the ultrafiltration procedure used for separating the different size-fraction of DOM affects the integrity of DOM (breaking up the size continuum), as indicated by differences in the optical indices direct values between CONTROL and H + L-DOM (Supplementary Table 2). We hypothesized that the disruption of low-energy bonds in gels and colloids during ultrafiltration (Verdugo, 2012) is one of the main reasons for the observed differences between these two treatments. Indeed, the resulting molecules might be more accessible to microbial degradation, as suggested by consistently higher prokaryotic abundance, % of live cells and leucine incorporation rates in H + L-DOM compared to CONTROL. Moreover, these bulk variables and the % of live cells were also larger in the H + L-DOM than in the L-DOM treatment, in agreement with previous studies in freshwater and marine systems (Benner and Amon, 2015). However, contrasting results have also been reported in coastal marine waters (Khodse and Bhosle, 2011).

The decrease of DOM molecular weight in the L-DOM treatment (observed by a higher increase of the s275-295 values; Helms et al., 2008), in parallel with a coupling to peak M and a254 (Figure 4), may indicate the ability of the assemblages growing on L-DOM to degrade older/more reworked DOM (Martínez-Pérez et al., 2017). Nevertheless, this coupling could also be related to the generation of refractory compounds (humic-like compounds, peak M) as subproducts of the remineralization processes (Jiao et al., 2010; Martínez-Pérez et al., 2017). Conversely, the H + L-DOM treatment was linked to peak T (protein-like compounds), pointing to a more important bond with the assimilation of labile material by the microbial communities growing on this niche. The fast turnover of this labile DOM (more variable and difficult to measure) may be the cause behind the major dispersion among the H + L-DOM replicates, compared to the CONTROL and the L-DOM replicates (Figure 4). Additionally, as pointed out by Yamashita and Tanoue (2003), some living organisms as bacteria display protein-like fluorescence. Since our samples were not filtered and they contain bacteria, the increase of this type of fluorescence during the incubations is more likely due to the increase of the microbial biomass.

In such a context, we must take into account that our study is based on “bulk measurements” that do not capture the material that is rapidly turned over (mostly within the low-molecular-weight fraction of DOM). Nevertheless, this rapidly consumed material could also be present in the dark ocean and sustain a select group of heterotrophic microbes in the deep ocean (Nagata et al., 2000; Hansell and Ducklow, 2003; Shen and Benner, 2020). Furthermore, compositional changes that occur during degradation are more complex than the simple removal of more labile compounds and the resultant accumulation of the remaining, less labile substrates. DOM reactivity also depends upon the consumer community composition. Thus, DOM is continually cycled and enzymatically hydrolyzed to smaller pieces (lower-molecular-weight compounds), which may likely persist at very low concentration. If so, the energetic gain from taking up these compounds may not pay off the cost of putting into operation the machinery to utilize them. Consequently, even intrinsically labile DOM may not be readily degraded due to limited availability or access to such molecules, according to the dilution hypothesis (Jannasch, 1967). This could provide a coherent explanation of why the DOM was relatively less reactive in the L-DOM treatment of our study, although there are still organisms potentially capable of degrading this relatively lower-molecular-weight DOM (see “Diversity and Community Composition Selection in Response to Size-Fractionated DOM Effect”). Alternatively, it is also likely that some DOM compounds are very resistant to microbial utilization due to their molecular properties (i.e., DOM quality). Rapid microbial utilization of diatom-derived DOM was recently observed in mesopelagic and deep waters of the North Atlantic, whereas marine humic substances were very resistant to utilization at elevated concentrations (Shen and Benner, 2020). The optical properties of the diatom –derived DOM were utilized within 48 h, whereas no substantial changes were observed in the optical properties of the humic substances. Labile forms of DOM are rapidly utilized by microorganism throughout the ocean water column, but elevated concentrations of marine humic substances are not. These observations indicate that not only DOM concentration but also DOM quality strongly influences its microbial utilization and fate in the ocean.

### Diversity and Community Composition Selection in Response to Size-Fractionated DOM Effect

The DOM quantity and quality of the water masses in the dark North Atlantic presented noticeable *in situ* variations in connection to the patterns of microbial (Bacteria and Archaea) communities (Guerrero-Feijóo et al., 2017). This previous evidence leads us to further hypothesize that different size-fractions of DOM will stimulate the growth of specific bacterial groups, which would be in turn linked to changes in the optical indices of the DOM. Our results showed a relatively narrow bacterial phylogenetic diversity in all treatments as compared to the original community, but still the reproducibility of our results across the three replicates per treatment supports the idea that these compositional changes were due to H + L- *versus* L-DOM effect (Figure 4). Indeed, changes in L- *versus* H + L-DOM utilization were likely driven by a few taxa within several families of Alpha-, Delta- and Gamma-proteobacteria, Bacteroidia, and Verrucomicrobia. However, the identity of the ASVs within these groups varied between the L- *versus* the H + L-DOM effect.

At the end of the incubation period, increased rates of leucine incorporation, bacterial abundance and% live cells in the H + L-DOM treatment co-occurred with the enrichment of a few families of Gammaproteobacteria, such as Shewanellaceae, Marinobacteraceae and Vibrionaceae (Figure 3 and Supplementary Table 3). Previous results from experimental incubations (Pinhassi and Berman, 2003; Nelson and Carlson, 2012; Balmonte et al., 2019; Reintjes et al., 2020), support the opportunistic/competitive life style of these Gammaproteobacteria, characterized by fast growth and a broad spectrum of active enzymes (Sebastián et al., 2018; Balmonte et al., 2019) leading to a wide metabolic potential (Lauro et al., 2009; Landa et al., 2013). Many of these copiotrophic bacteria are members of the rare biosphere (Sogin et al., 2006), underlining their potential for responding to sporadically environmental changes in the available organic matter, since they possess genetic repertories that enable a rapid exploitation of this material (Sogin et al., 2006), which could play an important role in environmental selection. For example, our investigation has shown that *Colwellia* sp. was represented by several ASVs which were unique in the H + L-DOM treatment. This response is not surprising, as *Colwellia* is regarded as a “boom and bust” specialist (Teira et al., 2008), highly responding to diatom-derived high-molecular-weight organic matter (Landa et al., 2018; Balmonte et al., 2019). Other genera showing unique ASVs in the H + L-DOM niche were *Burkholderia* sp., *Shewanella* sp. and *Vibrio* sp., which have been observed to predominate during bloom events (Allen et al., 2012; Sison-Mangus et al., 2016; Teeling et al., 2016; Bachmann et al., 2018). Besides, these genera were previously found at subsurface waters in our ecosystem area (Montes et al., 2020). We suggest that they could travel from surface associated to sinking particles (Mestre et al., 2018), particularly in the area of our study characterized by seasonal upwelling pulses, which support both the offshore export (Lønborg and Álvarez-Salgado, 2014; Lønborg et al., 2015) and sinking fluxes of organic matter (Teira et al., 2003; Álvarez-Salgado et al., 2006), and where an intense vertical mixing reaches down to the mesopelagic waters (Ruiz-Villarreal et al., 2006). This fact could have relevant consequences, because bacteria inhabiting these systems have the ability to function under extremely variable conditions (e.g., sporadically environmental changes determining the available organic matter), and thus likely play a disproportionately important role in the microbial-mediated cycling of marine nutrients.

A remarkable reproducibility in community composition was also observed among triplicate L-DOM replicates. Shifts in observed ASV richness were greatest with respect to the CONTROL than to H + L-DOM (Table 2), with a lower number of ASVs capable of thriving in the L-DOM niche, although they were distributed in a wider phylogenetic range (Flavobacteriaceae, Rhodobacteraceae, Oceanospirillaceae and Alteromonadaceae). The extent of these community shifts may be likely linked to the specific quality and quantity of the DOM compounds available in L-DOM treatment. As it was already mentioned, the dilution hypothesis could explain the lower reactivity of the DOM in the L-DOM treatment. Our investigation has found that still some taxa, presenting relatively low abundance in the original community, were enriched in the L-DOM treatment. These finding suggest that they are putatively capable of utilizing low-molecular-weight DOM compounds. For instance, Rhodobacteraceae family, which was among the significant prominent responders to L-DOM effect, is a commonly occurring member in natural open-ocean conditions, where labile DOM is scarce (Hansell, 2013). Specific ASVs within the Rhodobacteraceae family, such as *Loktanella* sp. and some other none identified members, were unique in L-DOM, which is not surprising as Rhodobacteraceae was shown to achieve significantly high turnover rates of low-molecular-weight compounds in the Atlantic waters (Alonso-Sáez et al., 2012). Similarly, AEGEAN 169 marine group (Alphaproteobacteria) was unique in L-DOM, in agreement with the recent findings of Zheng et al. (2020), showing that certain Alphaproteobacteria populations mainly utilized low-molecular-weight DOM due to the presence of specific transport systems for initial degradation of complex compounds. Moreover, some unique ASVs belonging to the genus *Oleiphilus* showed more than two-fold relative abundance in the L-DOM compared to the H + L-DOM treatment, in agreement with findings indicating that this genera can metabolized low-molecular-weight organic matter, including acetate and few carbohydrates (Sosa et al., 2017). Particularly interesting is the fact that the Order Flavobacteriales, although presenting ASVs in both treatments, displayed a much higher significant enrichment in the L-DOM than in the H + L-DOM niche. For example, some ASVs belonging to the genus *Tenacibaculum* (ASV6 and ASV9), a component of deep marine bacterial communities (Song et al., 2015), were unique in the L-DOM treatment. Bacteria belonging to *Tenacibaculum* are assumed to be important in the degradation of polymeric organic matter (Zheng et al., 2020) because of their ability to produce hydrolytic enzymes (Cottrell and Kirchman, 2000; Giovannoni and Rappé, 2000). Similarly, the genus *Aurantivirga* was associated to L-DOM treatment. Bacteria belonging to this genus were described in the deep ocean as chemoheterotrophic and aerobic bacteria (Song et al., 2015), and were found to be quite abundant during the course of incubations from natural blooms (Reintjes et al., 2020), when the supply of low-molecular-weight products from hydrolysis was larger. These evolving dominances of certain bacterial taxa depending on the different size-fraction incubations may likely imply that different microbial organisms may have diverse capabilities on the decomposition of DOM according to its molecular size.

## Conclusion

A 6-day incubation experiment of mesopelagic bacteria revealed a strong significant variation in abundance, activity and community composition of bacterial assemblages, along with shifts in the DOM composition, in response to size-fractionated natural DOM. In terms of bulk measurements, communities growing in the H + L-DOM showed an increase in cell number and activity compared to the L-DOM. Several members of Gammaproteobacteria preferentially utilized high-molecular-weight DOM, while some ASVs belonging to Flavobacteriales and Rhodobacterales (Alphaproteobacteria) thrived preferentially in the low-molecular-weight DOM. Specifically, the phylogenetic changes were a result of finely tuned bacterial response to L- *versus* H + L-DOM at ASV level. Furthermore, community response was accompanied by changes in the DOM composition (based on colored and fluorescent fractions of DOM) which follow microbial degradation. Our study have evidenced that microbial degradation of naturally occurring size-fractioned DOM have stimulated the selective growth of certain bacterial members, implying that different bacterial taxa may have different capabilities on degradation of DOM and thus providing insights into interactions of bacteria and organic matter in mesopelagic marine environments.

## Data Availability Statement

The datasets presented in this study can be found in online repositories. The names of the repository/repositories and accession number(s) can be found in the article/Supplementary Material.

## Author Contributions

MMV conceived the hypothesis and the objectives of this investigation, drafted the manuscript, and performed the analysis of flow cytometry and radio-isotopic methods on board. MMV and MN-C designed the experiment. EG-F and MMV carried out all biological measurements on board. Specifically, EG-F collected the DNA samples. MN-C prepared the treatments of dissolved organic matter and measured the DOM optical properties. TR-R performed the bioinformatics and the subsequently analysis of diversity data. All authors read, edited and approved the final manuscript.

## Conflict of Interest

The authors declare that the research was conducted in the absence of any commercial or financial relationships that could be construed as a potential conflict of interest.

## Footnotes

1 https://www.researchandtesting.com

2 http://www.xlstat.com
