# Supplementary material for: Changes in Activity and Community Composition Shape Bacterial Responses to Size-Fractionated Marine DOM
Source: Front Microbiol. 2020 Nov 20;11:586148. doi: 10.3389/fmicb.2020.586148 (PMC7714726; doi:10.3389/fmicb.2020.586148)
Supplement: Supplementary file 2 [file Data_Sheet_1.PDF]

Supplementary Materialfiles.

1 Supplementary Figures and Tables

1.1 Supplementary Figures

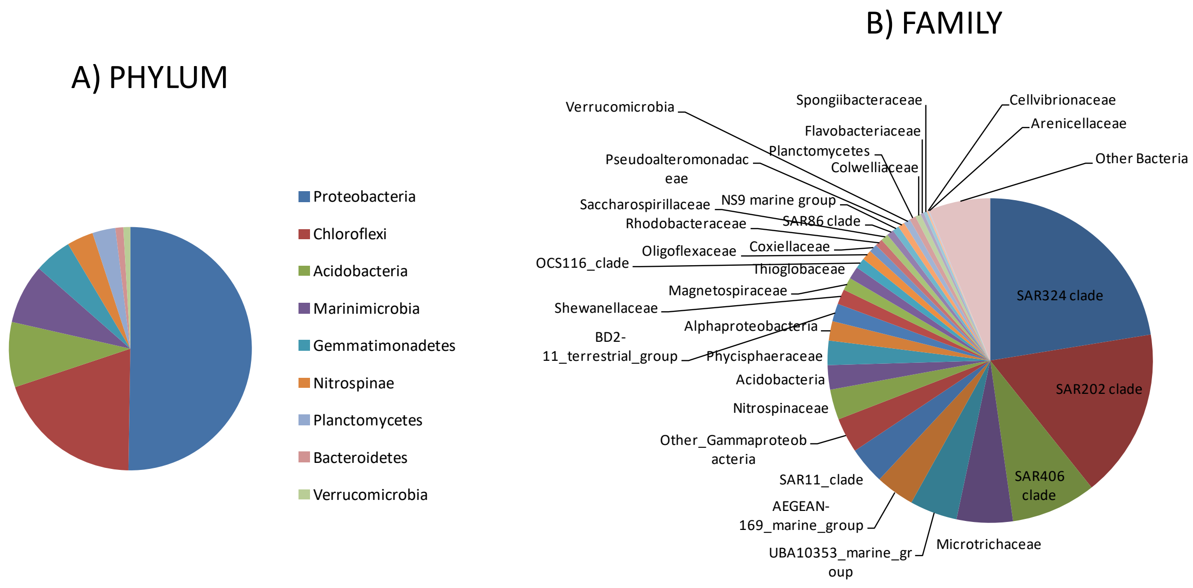

1.1.1. Supplementary Figure S1. Composition of the original bacterial community displaying (a) the relative distribution of the dominant phyla (B) taxonomic composition at the family level

1.2. Supplementary Tables

1.2.1. Supplementary Table S1. Increase/decrease in the average (triplicate replicates) concentration (µM) of nitrate + nitrite (NO_3^- + NO_2^-) and phosphate (PO_4^{3-}) at the end of the experiment with respect to the beginning of the experiment. The treatments were: 0.1 µm filtered seawater (CONTROL), 1 kDa filtered seawater, which is representative of low-molecular-weight DOM (L-DOM), and the combination of low- and high-molecular-weight DOM fractions in the original proportion (H+L-DOM). Three replicates were measured at each time point.

**Treatment**	**NO_3^- + NO_2^- (µM)**	**PO_4^{3-} (µM)**
CONTROL	-0.09 ± 0.03	-0.03 ± 0.02
H+L-DOM	-0.27 ± 0.09	-0.03 ± 0.02
L-DOM	0.18 ± 0.01	0.01 ± 0.04

1.2.2. Supplementary Table S2. Optical properties of the DOM of each replicate in the three treatments at the days 0 and 6. See abbreviations of each treatment in Figure 1. Abbreviations of optical properties: peak M, marine humic-like substances; peak T, protein-like substances; a254, absorption coefficient at 254 nm; a340, absorption coefficient at 340 nm; a365, absorption coefficient at 365 nm; s275-295, slope of the absorption spectrum between 275 and 295 nm. The treatments were: 0.1 µm filtered seawater (CONTROL), 1 kDa filtered seawater, which is representative of low-molecular-weight DOM (L-DOM), and the combination of low- and high- molecular-weight DOM fractions in the original proportion (H+L-DOM).

		**CONTROL**			**H+L-DOM**			**L-DOM**		
		**1**	**2**	**3**	**1**	**2**	**3**	**1**	**2**	**3**
peak M	T0	0.81 ± 0.03	0.82 ± 0.02	0.80 ± 0.01	0.86 ± 0.04	0.88 ± 0.05	0.82 ± 0.04	0.82 ± 0.03	0.85 ± 0.04	0.78 ± 0.04
(QSU)	T6	0.81 ± 0.03	0.85 ± 0.04	0.80 ± 0.01	0.87 ± 0.03	0.82 ± 0.06	0.83 ± 0.05	0.81 ± 0.02	0.80 ± 0.03	0.84 ± 0.03
peak T	T0	3.3 ± 0.2	3.5 ± 0.2	3.4 ± 0.3	5.2 ± 0.3	5.2 ± 0.2	5.3 ± 0.1	5.4 ± 0.2	5.3 ± 0.2	5.6 ± 0.1
(QSU)	T6	4.0 ± 0.4	4.4 ± 0.1	3.7 ± 0.4	6.4 ± 0.3	5.8 ± 0.2	6.1 ± 0.3	5.5 ± 0.2	6.0 ± 0.2	5.0 ± 0.2
a254	T0	0.96 ± 0.02	0.97 ± 0.02	0.96 ± 0.02	1.14 ± 0.02	1.08 ± 0.02	1.03 ± 0.02	0.95 ± 0.02	0.96 ± 0.02	0.98 ± 0.02
(m^{-1})	T6	0.93 ± 0.02	0.90 ± 0.02	0.91 ± 0.02	0.99 ± 0.02	0.97 ± 0.02	0.98 ± 0.02	0.90 ± 0.02	0.89 ± 0.02	0.90 ± 0.02
a340	T0	0.14 ± 0.02	0.15 ± 0.02	0.14 ± 0.02	0.18 ± 0.02	0.13 ± 0.02	0.10 ± 0.02	0.16 ± 0.02	0.16 ± 0.02	0.16 ± 0.02
(m^{-1})	T6	0.12 ± 0.02	0.12 ± 0.02	0.12 ± 0.02	0.10 ± 0.02	0.11 ± 0.02	0.13 ± 0.02	0.07 ± 0.02	0.08 ± 0.02	0.10 ± 0.02
a365	T0	0.09 ± 0.03	0.09 ± 0.03	0.09 ± 0.03	0.11 ± 0.03	0.08 ± 0.03	0.07 ± 0.03	0.08 ± 0.03	0.09 ± 0.03	0.07 ± 0.03
(m^{-1})	T6	0.07 ± 0.03	0.08 ± 0.03	0.08 ± 0.03	0.05 ± 0.03	0.06 ± 0.03	0.07 ± 0.03	0.04 ± 0.03	0.05 ± 0.03	0.06 ± 0.03
s275-295	T0	0.028 ± 0.000	0.028 ± 0.000	0.028 ± 0.000	0.027 ± 0.000	0.030 ± 0.000	0.032 ± 0.000	0.028 ± 0.000	0.028 ± 0.000	0.031 ± 0.000
	T6	0.028 ± 0.000	0.029 ± 0.000	0.029 ± 0.000	0.033 ± 0.000	0.032 ± 0.000	0.030 ± 0.000	0.032 ± 0.000	0.034 ± 0.000	0.032 ± 0.000

.

1.2.3. Supplementary Table S3. Presence/absence and phylogenetic assignation of the ASVs growing on CONTROL, H+L-DOM and L-DOM treatments. The treatments were: 0.1 µm filtered seawater (CONTROL), 1 kDa filtered seawater, which is representative of low-molecular-weight DOM (L-DOM), and the combination of low- and high- molecular-weight DOM fractions in the original proportion (H+L-DOM).

see attached excel file!

1.2.4. Supplementary Table S4. Results of the SIMPER analysis, showing the cumulative contribution (%) of individual ASVs to the dissimilarity found between L-DOM and H+L-DOM, explaining more than 50% of the dissimilarity, as well as between CONTROL and DOM-treatments.

Specie or ASV/comparison	L-DOM vs. H+L-DOM	CONTROL vs. H+L-DOM	CONTROL vs. L-DOM
Shimia (ASV23)	4.38	3.80	2.94
Shimia (ASV25)	3.74	3.17	1.06
Shewanella (ASV115)	3.63	1.69	5.40
Sacharospirillaceae (ASV183)	3.60	3.88	3.22
Shimia (ASV24)	3.21	3.04	1.96
Thalassotalea (ASV74)	2.69	2.23	0.85
Shewanella (ASV117)	2.64	2.49	-
Saccharospirillaceae (ASV184)	2.58	-	3.13
Shimia (ASV27)	2.55	1.88	1.61
Shewanella (ASV116)	2.29	2.15	-
Lentibacter /ASV12)	2.19	2.87	4.52
Oleiphilus (ASV151)	2.16	2.03	-
Vibrio (ASV213)	1.96	1.84	-
Shewanella (ASV118)	1.90	1.78	-
Lentibacter (ASV13)	1.86	1.07	2.13
Marinobacter (ASV86)	1.62	1.69	1.94
Thalassotalea (ASV75)	1.60	1.62	1.43
Shimia (ASV27)	1.58	1.66	2.05
Marinobacter (ASV27)	1.57	1.53	1.73
Thalassotalea (ASV73)	1.41	2.49	3.44
Marinobacter (ASV91)	1.34	1.25	-
Total	**50.49 %**	**44.17%**	**37.41%**
